# Antimicrobial Resistance and Virulence-Associated Traits of *Campylobacter jejuni* Isolated From Poultry Food Chain and Humans With Diarrhea

**DOI:** 10.3389/fmicb.2018.01508

**Published:** 2018-07-04

**Authors:** Kinga Wieczorek, Tomasz Wołkowicz, Jacek Osek

**Affiliations:** ^1^Department of Hygiene of Food of Animal Origin, National Veterinary Research Institute, Puławy, Poland; ^2^Department of Bacteriology and Biocontamination Control, National Institute of Public Health - National Institute of Hygiene, Warsaw, Poland

**Keywords:** *Campylobacter jejuni*, poultry food chain, humans, virulence genes, antimicrobial resistance, zoonotic pathogen

## Abstract

The objective of this study was to test the prevalence of virulence-associated markers and antimicrobial resistance in 624 *C. jejuni* isolated from poultry food chain, i. e., chicken feces (*n* = 160), poultry carcasses (*n* = 157), poultry meat (*n* = 152) and from humans (*n* = 155). All human strains were positive for 9 out of 13 putative virulence genes responsible for expression of pathogenic factors involved in different stages of the infection. The presence of all markers was also high in strains from chicken feces, carcasses and meat although not all of them were identified in 100% of the isolates. On the other hand, the *virB11, wlaN*, and *iam* putative pathogenic genes were detected in only 1.9, 15.2, and 20.5% of strains, respectively. *C. jejuni* isolates, irrespective of the origin, were highly resistant to ciprofloxacin (92.5% isolates), followed by nalidixic acid (88.9%) and tetracycline (68.4%). In case of ciprofloxacin, significantly more isolates from poultry feces, carcasses and meat were resistant than those obtained from humans and the same relationship was observed for tetracycline where the isolates from chicken feces were more often resistant than *C. jejuni* of carcasses and meat origin. A low number of strains was resistant to streptomycin (18.4% isolates) and only 5 strains (0.8%) displayed resistance to erythromycin. A relationship between resistance to fluoroquinolones and presence of selected pathogenic markers was observed, e.g., from 83.3% strains with the *virB11* to 93.4% with the *docA* genes were resistant to ciprofloxacin. The isolates that did not possess any of the pathogenic traits were also mainly resistant to this antimicrobial, although the number of such strains was usually low, except *virB11* (612 isolates), *wlaN* (529 strains), and *iam* (496 isolates). Furthermore, resistance to tetracycline was somehow associated with the presence of the virulence associated genes *wlaN* and *virB11* (56.8 and 75.0% isolates, respectively). The present study shows a high antimicrobial resistance to quinolones and tetracycline of *C. jejuni* isolated along poultry food chain and from patients with diarrhea, which was closely correlated with the presence of several virulence genes playing a role in the pathogenesis of *Campylobacter* infection.

## Introduction

*Campylobacter*, mainly *Campylobacter jejuni*, is one of the most common causes of foodborne bacterial infections worldwide (Allos, 2001; Bolton, 2015; Kaakoush et al., 2015; Tresse et al., 2017). Campylobacteriosis is also the most commonly reported zoonosis in the European Union (EU) with 246,307 confirmed cases and the notification rate 66.3 per 100,000 population in 2016 [EFSA (European Food Safety Authority) and ECDC (European Centre for Disease Prevention and Control), 2017]. The majority of the infections (83.6%) was caused by *C. jejuni* [EFSA (European Food Safety Authority) and ECDC (European Centre for Disease Prevention and Control), 2017]. The main transmission route of *Campylobacter* to humans is handling, preparation and consumption of contaminated food, especially of poultry origin (Allos, 2001; Park, 2002; Humphrey et al., 2007; Tresse et al., 2017). *C. jejuni* does not cause clinical diseases in poultry, but poultry carcasses have been frequently contaminated in the slaughterhouse due to a high prevalence of these bacteria in the intestinal tract of chickens; therefore, poultry carcasses can serve as the source of these microorganisms to humans (Humphrey et al., 2007; van Gerwe et al., 2010). Although mortality in humans is low, morbidity due to post-infectious sequelae such as Guillain-Barré syndrome, reactive arthritis and irritable bowel syndrome is significant (Allos, 2001; Humphrey et al., 2007; Iovine, 2013; Tresse et al., 2017).

The pathogenesis of *Campylobacter* infection is complex and still poorly understood. However, it is believed that the expression of genes involved in motility, colonization, epithelial cell invasion, and toxin production play an important role in the disease development (Humphrey et al., 2007; Dasti et al., 2010). Mobility of the bacterial cells, involving the coordination of several genes (i.e., *flaA* and *flhA*), is essential for passage through the stomach and gut environment (Park, 2002) where *Campylobacter* produces several cell-surface proteins (encoded by the *cadF, docA, racR, virB11, ciaB*, and *iam* genes) that promote to adhere to and invade intestinal epithelial cells (Konkel et al., 1997; Carvalho et al., 2001; Dasti et al., 2010). The bacteria can also excrete several cytotoxins (encoded by the *cdtA, cdtB, cdtC, wlaN* genes) that contribute to the development of the disease (Hickey et al., 2000; Tresse et al., 2017). Furthermore, *C. jejuni* is able to produce superoxide dismutase enzyme (encoded by the *sodB* marker), which catalyzes the breakdown of superoxide radicals and it is one of the bacterial major defense mechanisms against oxidative damage (Pesci et al., 1994).

There has been an increasing trend of antimicrobial resistance in *Campylobacter* isolated within the food chain and humans in recent years (Melero et al., 2012; Piccirillo et al., 2013; Wieczorek et al., 2013b; Ma et al., 2014; Abdollahpour et al., 2015). Macrolides (i.e., erythromycin) and fluoroquinolones (i.e., ciprofloxacin) are considered as the first- and second-choice of antimicrobials, respectively for the treatment of human *Campylobacter* infections (Ge et al., 2013; Iovine, 2013). Most campylobacteriosis cases are usually self-limiting and do not require hospitalization and antimicrobial treatment. However, the therapy is required in children with fever, increasing bloody diarrhea, and in eldery or immunocompromised patients with severe and prolonged systemic disorders (Allos, 2001; Ge et al., 2013; Tresse et al., 2017). In these cases, macrolides (mostly erythromycin and azithromycin) are usually the first-choice antibiotics whereas fluoroquinolones and, to a less extent, tetracycline are alternative options (Allos, 2001; Iovine, 2013). A significant use of antimicrobials in animals and in humans has leaded to an increase in antibiotic-resistant *Campylobacter* population (Humphrey et al., 2007; Ge et al., 2013). Thus, monitoring of *C. jejuni* resistance is highly relevant to public health.

The objective of this study was to investigate the prevalence of virulence-associated genes and antimicrobial resistance in *C. jejuni* isolated from poultry food chain and from humans.

## Materials and methods

### Collection of *C. jejuni* isolates

Sampling and *Campylobacter* isolation from chickens was performed in years 2014 and 2016 using the procedure as described earlier (Wieczorek et al., 2013b). Briefly, intact ceca from 10 birds were taken after evisceration, the content was pooled and one loop-full (10 μl) of the material was streaked directly on Karmali agar (*Campylobacter* Agar Base + *Campylobacter* Supplement; Oxoid, UK) and *Campylobacter* blood-free agar (Oxoid) with CCDA selective supplement (Oxoid) and incubated at 41.5°C ± 1°C for at least 48 h ± 2 h in a microaerobic atmosphere generated using CampyGen kit (Oxoid). *Campylobacter* from poultry feces was isolated by direct plating on two agar plates (Karmali and *Campylobacter* blood-free) as described above. After incubation, the plates were examined for morphologically typical *Campylobacter* colonies (grayish, often with a metallic sheen, flat and moist with a tendency to spread) and colony identity was confirmed by microscopic examination of morphology and motility, microaerobic growth at 25°C, and the presence of oxidase. From each fecal sample one presumptive *Campylobacter* isolate was then confirmed by PCR as described previously (Wieczorek et al., 2013b). A total of 160 confirmed *C. jejuni* isolates were used for the present analyzis.

The swab samples were collected directly after immersion chilling (0–4°C) but before further processing from the neck skin and the skin surface under the wings of the broiler carcasses and immediately transported to the laboratory in Amies transport medium with charcoal (Medlab, Poland). *Campylobacter* bacteria were isolated as described (Wieczorek et al., 2013a). Briefly, the swabs were placed in 5 ml of Bolton enrichment broth (Oxoid) supplemented with vancomycin, cefoperazone, trimethoprim, and amphotericin B and incubated as above. The cultures were then plated onto Kamali agar (Oxoid) and *Campylobacter* blood-free agar with CCDA selective supplement (Oxoid) and incubated at 41.5°C ± 1°C for 48 h ± 2 h under microaerobic conditions. From each sample one presumptive *Campylobacter* isolate was confirmed using PCR as described previously (Wieczorek et al., 2013b). During 5 years (2012–2016) a total of 157 confirmed *C. jejuni* were collected for the current investigation.

The *Campylobacter* strains from chicken meat (*n* = 152) were recovered in years 2010–2012 and 2015–2016 using the ISO 10272-1 standard and *C. jejuni* isolates were confirmed with the PCR method as described for the broiler carcasses.

A total of 155 *C. jejuni* isolates were obtained during the period of 2011–2016 from patients with diarrhea using standard culturing techniques. Rectal swabs were directly streaked onto mCCDA agar (Oxoid) and incubated at 41.5°C ± 1°C for 48 h ± 2 h under microaerobic conditions to grow only thermophilic campylobacters. Then, typical *Campylobacter* colonies were selected for further investigation using standard biochemical tests *C. jejuni* was identified with PCR as described previously (Vandamme et al., 1997).

Altogether, 624 *C. jejuni* were isolated and stored at −80°C until further analyzes.

### Presence of virulence factor genes

*Campylobacter* isolates were tested for the presence of the following virulence genes: *flaA* and *flhA* (involved in motility), *cadF, docA, racR, virB11* (responsible for adhesion and colonization) *cdtA, cdtB, cdtC, wlaN* (cytotoxin production). Additionally, the gene markers such as *ciaB* and *iam* responsible for the invasiveness of *Campylobacter*, and *sodB* (stress response) were also amplified. The PCR primer sequences and annealing temperatures are shown in Table S1.

### Antimicrobial resistance testing

A microbroth dilution method was used to establish the minimum inhibitory concentrations (MICs) of *C. jejuni* isolates to 6 antimicrobials (gentamicin, streptomycin, erythromycin, ciprofloxacin, nalidixic acid, and tetracycline) using the Sensititre® custom susceptibility plates, EUCAMP (Trek Diagnostics, UK). The strains were sub-cultured twice on Columbia agar (Oxoid) at 41.5°C for 48 h under microaerobic conditions. The minimum inhibitory concentration of the antimicrobial agents was determined using Mueller-Hinton Broth (Oxoid) supplemented with 2–2.5% horse blood (Trek). The plates were incubated at 37°C for 48 h under microaerophilic conditions and read using the Vision® system (Trek). The obtained results were determined according to the established breakpoints (Table S2). The antimicrobials and cut off values used for the interpretation of the MIC results were in accordance with EUCAST (Sifr et al., 2015) and the European Union Reference Laboratory for Antimicrobial Resistance.

### Statistical analysis

The chi-square test with Yates' correction was used to examine differences in prevalence of virulence marker genes and antimicrobial resistance of *C. jejuni* isolated from different sources as well as to identify associations between antimicrobial resistance and presence of virulence marker genes. *P* < 0.05 was considered as significant. Confidence intervals (CIs) with 95% of confidence level in resistant strains were also calculated.

## Results

### Prevalence of virulence genes

Overall, the presence of virulence marker genes among analyzed *C. jejuni* isolates was high, especially for the genes associated with motility, adhesion and colonization, (except the *virB11* marker), cytotoxin production (except *wlaN*), and invasiveness (except *iam*) of the bacteria (Table 1). In case of human isolates, all of them were positive for 9 out of 13 putative virulence gene markers tested, i.e., *flaA, cadF, docA, racR, cdtA, cdtB, cdtC, ciaB*, and *sodB*. The vast majority of such strains (99.4%) were also positive for the *flhA* gene. The presence of all these markers was also high in strains from chicken feces, carcasses and meat although not all of them were identified in 100% of the isolates (Table 1). On the other hand, a low prevalence of the putative pathogenic marker genes was associated with the *virB11* which was detected in only 12 (1.9%) of the total number of strains. This gene was not identified among *C. jejuni* of poultry meat origin and in only one isolate recovered from poultry feces. Furthermore, the *wlaN* and *iam* genes were found in 15.2 and 20.5% of all strains tested, respectively (Table 1).

**Table 1:** Distribution of virulence associated and environmental resistance genes in *C. jejuni* tested.

**Virulence trait**	**Gene**	**Percentage of positive isolates**				
		**Chicken feces**	**Poultry carcasses**	**Poultry meat**	**Human feces**	**Combined**
		**(*n* = 160)**	**(*n* = 157)**	**(*n* = 152)**	**(*n* = 155)**	**(*n* = 624)**
Motility	flaA^*^	98.1	98.7	98.7	100	98.9
	flhA^*^	99.4	98.1	99.3	99.4	99.0
Adhesion and colonization	cadF^*^	99.4	98.7	100	100	99.5
	docA^*^	100	98.1	96.7	100	98.7
	*racR*	100	95.5	96.0	100	97.9
	*virB11*	0.6	2.5	0	4.5	1.9
Cytotoxin production	*cdtA*	94.4	96.2	96.0	100	96.6
	*cdtB*	94.4	98.1	94.1	100	96.6
	*cdtC*	96.2	96.8	97.4	100	97.6
	wlaN^*^	13.7	17.2	12.5	17.4	15.2
Invasiveness	ciaB^*^	99.4	96.8	97.4	100	98.4
	*iam*	26.2	8.9	31.6	15.5	20.5
Stress response	sodB^*^	98.1	100	99.3	100	99.4

* *No statistically significant differences between presence of virulence marker genes among different sources of the isolates have been identified. The following differences were identified for the presence of: cdtA from poultry carcasses and human feces (P < 0.05), from chicken and human feces (P < 0.01); cdtB from poultry and human feces (P < 0.01), from poultry meat and human feces (P < 0.01); cdtC from chicken and human feces (P < 0.05); virB11 from poultry meat and human feces (P < 0.05); racR from poultry feces and carcasses (P < 0.05), poultry carcasses and human feces (P < 0.05), poultry feces and meat (P < 0.05), poultry meat and human feces (P < 0.05); iam from poultry feces and carcasses (P < 0.001), poultry feces and meat (P < 0.0001), poultry and human feces (P < 0.05), poultry meat and human feces (P < 0.01)*.

### Antimicrobial resistance

The results of antimicrobial resistance of the *C. jejuni* isolates are shown in Table 2. Overall, most of the strains were resistant to ciprofloxacin (total 577; 92.5% isolates), nalidixic acid (555; 88.9%) and, in a less extent, to tetracycline (427; 68.4%). There were statistical differences in the resistance rates between strains recovered from poultry chain and humans. In case of CIP significantly more isolates from poultry feces, carcasses and meat were resistant than those obtained from human patients (*P* < 0.005, *P* < 0.05, and *P* < 0.005, respectively). The same relationship was observed for tetracycline where the isolates from chicken feces were more often resistant than *C. jejuni* of carcasses and meat origin (*P* < 0.05 and *P* < 0.0001, respectively). A low number of isolates, irrespective of the origin, was resistant to streptomycin (115; 18.4% isolates) and higher resistance rates was observed among strains from chicken feces compared to the isolates from meat (*P* < 0.001) and humans (*P* < 0.0001). It was also found that only 5 of 624 strains (0.8%) displayed resistance to erythromycin (Table 2).

**Table 2:** Antimicrobial resistance of *C. jejuni* isolated from different sources. Values are in % ± 95% CI.

**Antimicrobial**	**Percentage of resistant isolates**							
	**Chicken feces (*****n*** = **160)**		**Poultry carcasses (*****n*** = **157)**		**Poultry meat (*****n*** = **152)**		**Human feces (*****n*** = **155)**	
	**%**	**95% CI**	**%**	**95% CI**	**%**	**95% CI**	**%**	**95% CI**
Ciprofloxacin (CIP)	95.0	3.38–3.38	93.6	3.82–3.82	96.1	3.10–3.10	85.2	5.60–5.60
Nalidixic acid (NAL)	83.1	5.80–5.80	92.4	4.16–4.16	96.1	3.10–3.10	84.5	5.70–5.70
Streptomycin (STR)	21.0	6.37–6.37	21.0	6.37–6.37	12.5	5.26–5.26	10.3	4.79–4.79
Erythromycin (ERY)	0.6	0.63–1.22	0.6	0.63–1.22	1.3	1.32–1.81	0.6	0.63–1.22
Tetracycline (TET)	78.1	6.41–6.41	66.9	7.36–7.36	57.9	7.85–7.85	70.3	7.20–7.20

*CI, confidence intervals with 95% confidence level*.

The MICs distribution of all 624 *C. jejuni* isolates tested is shown in Table 3. Among strains resistant to ciprofloxacin (total 577 isolates), several demonstrated a high resistance rates showing the MIC values ≥16 mg/L (229 strains; 39.7%). The majority of such isolates was recovered from chicken feces (84 out of 152; 55.3%) whereas only 38 of 132 (28.8%) highly resistant strains were isolated from humans (Table 3). A very high resistance rate was observed for tetracycline (total 427 isolates) where 395 (92.5%) displayed the MIC values ≥64 mg/L. These highly resistant strains were recovered from all sources, i.e., poultry ceca (94.4% isolates), carcasses (98.1%), meat (89.8%), and humans (96.3%). Almost all isolates resistant to streptomycin (*n* = 115 strains) showed a high resistance pattern manifested with MICs ≥16 mg/l (total 114; 99.1% strains). The majority of these *C. jejuni* (93; 81.6% isolates) had MIC above 16 mg/L and they were recovered from all but poultry meat sources (Table 3). A very few strains were resistant to erythromycin (a total of 5 isolates); however, 4 of them demonstrated a high MIC values (≥128 mg/L). These strains were only isolated along poultry meat chain (Table 3).

**Table 3:** Antimicrobial resistance and distribution of MICs among *C. jejuni* isolated from different origins.

**Number of isolates with MICs (mg/L)**																			
**Antimicrobial**	**Origin**	**No. of resistant strains**	≤**0.12**	≤**0.25**	**0.25**	≤**0.5**	**0.5**	≤**1**	**1**	**2**	**4**	**8**	**16**	>**16**	**32**	**64**	>**64**	**128**	>**128**
CIP	Feces	152	8							1	10	57	31	53					
	Carcasses	147	9		1					1	13	76	22	35					
	Meat	146	6						2	2	23	69	33	17					
	Human	132	22		1						9	85	21	17					
NAL	Feces	133								13	9	3	2		3	21	109		
	Carcasses	145								4	8					36	109		
	Meat	146								3	5				14	19	111		
	Human	131								2	20	2		131					
STR	Feces	47		2			16		73	21	1	1	1	45					
	Carcasses	33		4			4		77	38	1		1	32					
	Meat	19		2			16		93	21	1		19						
	Human	16					2		95	41	1			16					
ERY	Feces	1						157		1	1								1
	Carcasses	1						155		1						1			
	Meat	2						146		2	1							1	1
	Human	1						154											1
TET	Feces	125				33			2	2			1		4	21	97		
	Carcasses	105				48	2		2	2			2		8	23	70		
	Meat	88				61			3	2			2		5	18	61		
	Human	109					45		1	3					1	33	72		
GEN	Feces	0	15		58		83		4										
	Carcasses	0	12		78		60		7										
	Meat	0	16		90		45		1										
	Human	0	1		52		99		3										

*Cut-off values are marked as vertical lines*.

### Association between virulence genes and antimicrobial resistance

Table 4 shows the prevalence of each virulence gene among all 624 *C. jejuni* isolates that were either resistant or sensitive to ciprofloxacin, nalidixic acid, streptomycin or tetracycline. The vast majority of strains resistant to CIP or NAL were positive for virulence markers tested, between 83.3% with the *virB11* gene (only 12 such isolates identified) to 93.4% with the *docA* marker. On the other hand, the isolates that did not possess any of the pathogenic genes were also mainly resistant to these two antimicrobials, although the number of such strains was usually low, except *virB11* (612 isolates), *wlaN* (529 strains), and *iam* (496 isolates). No statistical differences were identified among isolates resistant to ciprofloxacin and in *C. jejuni* resistant to NAL such differences were detected in strains positive and negative for the *cdt* toxin genes.

**Table 4:** Relationship between virulence genes and antimicrobial resistance patterns in all *C. jejuni* tested.

**Virulence gene**	**Number (percentage) of isolates resistant to:**			
	**CIP (*****n*** = **577)**	**NAL (*****n*** = **555)**	**STR (*****n*** = **115)**	**TET (*****n*** = **427)**
*flaA*^+^ (*n* = 617) *flaA*^−^ (*n* = 7)	570 (92.4) 7 (100)	548 (88.8) 7 (100)	114 (18.5) 1 (14.3)	425 (68.9)^*^ 2 (28.6)
*flhA*^+^ (*n* = 618) *flhA*^−^ (*n* = 6)	571 (92.4) 6 (100)	549 (88.9) 6 (100)	115 (18.6) 0	425 (68.8) 2 (33.3)
*cadF*^+^ (*n* = 631) *cadF*^−^ (*n* = 3)	574 (91.0) 3 (100)	552 (87.5) 3 (100)	115 (18.2) 0	425 (67.3) 2 (66.7)
*docA*^+^ (*n* = 616) *docA*^−^ (*n* = 8)	569 (93.4) 8 (100)	547 (88.8) 8 (100)	113 (18.3) 2 (25.0)	421 (68.3) 6 (75.0)
*racR*^+^ (*n* = 611) *racR*^−^ (*n* = 13)	565 (92.5) 12 (92.3)	543 (88.9) 12 (92.3)	115 (18.8) 0	421 (68.9) 6 (46.1)
*virB11*^+^ (*n* = 12) *virB11*^−^ (*n* = 612)	10 (83.3) 567 (92.6)	10 (83.3) 545 (89.0)	0 0	9 (75.0) 418 (68.3)
*cdtA*^+^ (*n* = 603) *cdtA*^−^ (*n* = 21)	556 (92.2) 21 (100)	542 (89.9)^***^ 13 (61.9)	104 (17.2)^***^ 11 (52.4)	411 (68.2) 16 (76.2)
*cdtB*^+^ (*n* = 603) *cdtB*^−^ (*n* = 21)	556 (92.2) 21 (100)	541 (89.7)^***^ 14 (66.7)	106 (17.6)^**^ 9 (42.9)	414 (68.7) 13 (61.9)
*cdtC*^+^ (*n* = 609) *cdtC*^−^ (*n* = 15)	562 (92.3) 15 (100)	546 (89.6)^***^ 9 (60.0)	109 (17.9)^*^ 6 (40.0)	417 (68.5) 10 (66.7)
*wlaN*^+^ (*n* = 95) *wlaN*^−^ (*n* = 529)	87 (91.6) 490 (92.6)	87 (91.6) 468 (88.5)	6 (6.3)^***^ 109 (20.6)	54 (56.8)^**^ 373 (70.5)
*ciaB*^+^ (*n* = 614) *ciaB*^−^ (*n* = 10)	567 (92.3) 10 (100)	545 (88.8) 10 (100)	114 (18.6) 1 (10.0)	421 (68.6) 6 (60.0)
*iam*^+^ (*n* = 128) *iam*^−^ (*n* = 496)	118 (92.2) 459 (92.5)	115 (89.8) 440 (88.7)	15 (11.7) 100 (20.2)	83 (64.8) 344 (69.3)
*sodB*^+^ (*n* = 620) *sodB*^−^ (*n* = 4)	573 (92.4) 4 (100)	551 (88.9) 4 (100)	113 (18.2) 2 (50.0)	425 (68.5) 2 (50.0)

CIP, ciprofloxacin; NAL, nalidixic acid; STR, streptomycin; TET, tetracycline;

* P < 0.05;

** P < 0.01;

*** *P < 0.001*.

*C. jejuni* resistant to tetracycline were also associated with many of the virulence genes identified although such correlation was not as strongly expressed as for CIP and NAL. The percentage of strains resistant to TET and positive for the pathogenic markers was from 75.0% (*virB11* gene; only 12 positive isolates) and 68.9% (*flaA* and *racR* genes) to 56.8% (*wlaN* marker). Statistically significant differences were only observed between the isolates with/without the *flaA* and *wlaN* markers (Table 4).

## Discussion

The present study provides the results on analysis of the prevalence putative gene markers and antimicrobial resistance among *C. jejuni* isolates along poultry food chain and humans with diarrhea. The genes associated with bacterial motility (*flaA* and *flhA*) and adhesion to epithelial cells (*cadF, docA*, and *racR*), which are the key mechanisms in the development of *Campylobacter* infection, were identified in most or even all isolates from the analyzed sources, especially from persons suffering from campylobacteriosis (Allos, 2001; Humphrey et al., 2007; Tresse et al., 2017). These findings provide further evidence that flagellar and adhesion genes are highly conserved among *C. jejuni* as previously suggested by several authors (Datta et al., 2003; Müller et al., 2006; Thakur et al., 2010; Koolman et al., 2015; Lapierre et al., 2016). Only few isolates (1.9% in total) were positive for the *virB11* gene encoding a putative type IV secretion system involved in adherence of campylobacters to the gut epithelial cells (Bacon et al., 2000). Most of the *virB11*-positive isolates were identified in the current investigation among human *C. jejuni* which may suggest the role of this marker in pathogenesis of the diarrhea. There are also information that this gene is more often absent in human isolates and therefore may not be involved in virulence and pathogenesis of campylobacteriosis (Datta et al., 2003; Müller et al., 2006; Talukder et al., 2008).

Several strains were negative for the *wlaN* gene responsible for the production of β-1,3 galactosyltransferase involved in cell wall synthesis (only 15.2% positive isolates) but the absence of this marker has been previously observed (Datta et al., 2003; Talukder et al., 2008; Koolman et al., 2015). On the other hand, Kim et al. (2016) identified the *wlaN* gene among 100% of 63 human and in 78.6% of 42 animal *C. jejuni* isolated tested in Korea. The product of the *wlaN* gene shows ganglioside mimicking structures and thus may be involved in developing of Guillain–Barre' syndrome after *C. jejuni* infection (Thakur et al., 2010; Kim et al., 2016; Lapierre et al., 2016).

Other often prevalent virulence marker determinants included *cdtA, cdtB*, and *cdtC* cytotoxin genes which cause an important role in diarrhea by interfering with the division and differentiation of the intestinal crypt cells (mean prevalence of 96.6–97.6% positive strains). As it has been shown in previous investigations all three subunits are required for full toxin activity (Park, 2002; Lapierre et al., 2016). Interestingly, all 155 human *C. jejuni* isolates tested were positive for three cytotoxin subunit genes. However, some strains of poultry origin were negative for one or two subunit determinants which may suggest that they were not able to express the entire product or the toxin genes were not identified with the primers used in the study due to e.g., point mutations in the coding region (Bang et al., 2004).

Other genes involved in stress response and invasiveness, which are important for *Campylobacter* survival in the intestinal tract (*sodB* and *ciaB*), were in a high prevalence among all strains analyzed in the current study (99.4 and 98.4% of the isolates; including all positive *C. jejuni* of human origin). The product of the *ciaB* marker, which play a role both in the invasiveness and in colonization of the epithelial cells, was identified in campylobacters by other authors either in a lower percentage (Ziprin et al., 2001; Hanning et al., 2010) or in similar one to the present study (Raeisi et al., 2017). Since the *ciaB* and *sodB* genes are important in the initial stages of colonization, the high prevalence of these markers in *C. jejuni* currently tested, especially among strains isolated from humans with diarrhea, may suggest that these bacteria were able to overcome the stress conditions during passage through the intestinal tract and then induce the disease.

*C. jejuni* isolates tested, regardless the origin, were most frequently resistant to quinolones (ciprofloxacin and nalidixic acid; 92.5 and 88.9% of total isolates, respectively). A total of 39.7 and 76.0% of ciprofloxacin- and nalidixic acid-resistant *C. jejuni* investigated had the MIC values ≥16 mg/l and ≥32 mg/l, respectively. The cause of such high resistance to quinolones of strains isolated along chicken-production chain could be related to a broad use of enrofloxacin in veterinary medicine, especially in poultry production, and are thought to play a role in the spread of resistance to human isolates (Griggs et al., 2005; Iovine, 2013). A high rate of resistance to quinolones has also been reported previously in Poland, both among isolates of poultry and human origins (Wardak et al., 2007; Wieczorek et al., 2013b; Andrzejewska et al., 2015; Szczepanska et al., 2017; Wozniak-Biel et al., 2018). Data described in the recent EFSA/ECDC antimicrobial resistance report for 2016 have shown that *C. jejuni* isolated from humans were in average in 54.6% resistant to ciprofloxacin (information from 17 countries; no data from Poland) [EFSA (European Food Safety Authority) and ECDC (European Centre for Disease Prevention and Control), 2018]. Even higher resistance rate to ciprofloxacin was noted for *C. jejuni* isolated from broiler meat (mean value 64.9% strains; data from only 6 EU Member States) but Poland has not provided such information. On the other hand, the vast majority of the isolates from broilers displayed resistance to ciprofloxacin (93.2%) which was much higher than the EU mean rate (66.9%) [EFSA (European Food Safety Authority) and ECDC (European Centre for Disease Prevention and Control), 2018]. Such high percentage of *C. jejuni* resistant to quinolones in Poland may be due to a broad use of these antimicrobials in animal husbandry. According to the recent European Medicines Agency report, in Poland from 2011 to 2015 an increase was observed in sales (in mg for population correction unit, PCU) of fluoroquinolones used in veterinary medicine (EMA, 2017). In 2011, the proportion of total sales for fluoroquinolones was 5.7% whereas this figure in 2015 was 6.2%. In 2015, the sales of fluoroquinolones were 8.56 mg/PCU, while average value for 25 European countries described in the report in that year were 2.75 mg/PCU. Fluoroquinolones are considered by the World Health Organization as critical drugs for the treatment of humans, therefore investigation of *Campylobacter* resistance to these antimicrobials in food-producing animals is important for the public health. A high resistance to ciprofloxacin has been reported among human *C. jejuni* isolates in Korea (96.8%), China (93.1%), Qatar (63.2%), Estonia (67.9%), international travelers (from 50.8% in Africa to 75.0% in Asia), and the United Arab Emirates (85.4%) (Sonnevend et al., 2006; Unicomb et al., 2006; Kim et al., 2016; Zhou et al., 2016; Post et al., 2017). Strains from other countries have shown lower rates of resistance, e.g., 30.5% in Canada, 8.4% in Finland, 2% in Australia, and between 0 and 9% in Sweden (Osterlund et al., 2003; Ghunaim et al., 2015; Riley et al., 2015; Olkkola et al., 2016). It has been considered that the absence or low prevalence of *C. jejuni* ciprofloxacin-resistant isolates in some countries has been attributed previously to restricting the use of fluoroquinolones in food-producing animals (Osterlund et al., 2003; Griggs et al., 2005; Unicomb et al., 2006).

Many isolates displayed resistance to tetracycline (68.4%), especially *C. jejuni* recovered from chicken feces (78.1%) but also from humans with diarrhea (70.3%). It may suggest that poultry can serve as important reservoir of such strains for humans. Recent studies also indicated that *C. jejuni* of poultry sources were often resistant to tetracycline at the rates from 32.3% of the isolates in Chile (Lapierre et al., 2016), 75.5% in Iran (Raeisi et al., 2017), 79.4% in China (Han et al., 2016) up to 83.5% in the USA (Ladely et al., 2017). Investigations performed earlier in Poland showed different resistance levels to this antimicrobial, ranging from 9.0% (Rozynek et al., 2008), 31.1% (Wieczorek et al., 2015), 42.3% (Andrzejewska et al., 2015), 46.5% (Wieczorek and Osek, 2015), 51.1% (Szczepanska et al., 2017) up to even 100% of *C. jejuni* tested (Wozniak-Biel et al., 2018). Human *C. jejuni* isolates resistant to tetracycline were also identified during studies in several countries where the percentage of positive strains was from 2.1% (Olkkola et al., 2016), 24.3% (Lapierre et al., 2016), 42.9% (Mäesaar et al., 2016), 48.3% (Post et al., 2017), 64.4% (Riley et al., 2015) to 74.6% (Kim et al., 2016). Furthermore, such resistant bacteria were also previously detected in Poland among isolates from patients with diarrhea at the constantly increasing level still lower that identified in the current investigation, i.e., 13.7% (Wardak et al., 2007), 17.5% (Rozynek et al., 2009), 39.1% (Szczepanska et al., 2017) and up to 40% (Wardak and Szych, 2010). Recent results from EFSA/ECDC report show that 42.8% of *C. jejuni* from humans isolated in the European Union were resistant to tetracycline (lack information from Poland) [EFSA (European Food Safety Authority) and ECDC (European Centre for Disease Prevention and Control), 2018]. Similar resistance levels were observed for the isolates from broiler meat (48.6%) and broilers (50.7%). However, the last value was much higher in Poland (71.6% of resistant strains). This can be the result of a broad use of tetracyclines in veterinary medicine at the level of 42.9 mg/PCU (EMA, 2017).

Erythromycin is the drug of choice for the treatment of *C. jejuni* infections and resistance levels observed in all isolates regardless the origin were very low (0.8% resistant strains in total). The percentage of macrolide-resistant isolates recovered previously from poultry chain was usually also low but higher than obtained in the present investigation, and ranged from 2.2 to 26.0% (Han et al., 2016; Lapierre et al., 2016; Mäesaar et al., 2016; Ladely et al., 2017; Raeisi et al., 2017). Analyzes of such isolates in Poland demonstrated that erythromycin resistant levels among *C. jejuni* were rather low, from 0% (Wieczorek et al., 2013b; Wieczorek and Osek, 2015; Wozniak-Biel et al., 2018), 2.4% (Wieczorek et al., 2015), 3.0% (Andrzejewska et al., 2015) to 3.3% (Szczepanska et al., 2017). At the EU level, the percentage of such resistant strains in 2016 was 2.2% for broiler meat and 1.3% (0% in Poland) for broilers [EFSA (European Food Safety Authority) and ECDC (European Centre for Disease Prevention and Control), 2018].

In the present study only one isolate (0.6%) of human origin displayed resistance to erythromycin which was a lower rate than identified in similar strains by other authors: 1.5% (Lapierre et al., 2016; Cha et al., 2017), 3.9% (Riley et al., 2015), 4.8% (Kim et al., 2016) and 8.6% (Ghunaim et al., 2015). Recent EFSA/ECDC report demonstrated that mean European level of *C. jejuni* resistance to erythromycin was 2.1% out of 21,993 isolates tested (no data from Poland) [EFSA (European Food Safety Authority) and ECDC (European Centre for Disease Prevention and Control), 2018]. There are not many results concerning resistance to macrolides of *C. jejuni* from human patients in Poland; however, the previous investigations demonstrated that either all isolates tested were sensitive to azithromycin and/or erythromycin (Wardak et al., 2007; Szczepanska et al., 2017) or only a low percentage displayed resistance to erythromycin, i.e., 0.4 and 1.7% strains (Rozynek et al., 2008, 2009).

Analysis of relationship between the presence of putative virulence genes and antimicrobial resistance of *C. jejuni* isolates did not show clear correlations. The percentages of strains with pathogenic factor that were either resistant or sensitive to antimicrobials tested were similar, although some statistical differences were identified, especially among isolates resistant to streptomycin with and without the *cdt* and *wlaN* genes responsible for cytotoxin production. Furthermore, single strains negative for the *flhA, cadF*, and *racR* markers were sensitive for streptomycin but the number of such isolates were very low to draw any conclusions. We have demonstrated that *C. jejuni* with the virulence markers tested were mostly resistant to ciprofloxacin, which is used for treatment of humans with campylobacteriosis or in patients with presumed *Campylobacter* infections not confirmed by laboratory analyzes. Positive and negative associations between virulence genes and antimicrobial resistance have been previously identified in other bacterial pathogens (McGowan-Spicer et al., 2008; Adib et al., 2014). It seems that the presence of antimicrobial resistance and potential virulence factors are both important for development of the disease. Therefore, further investigation on the interactions between virulence markers and antimicrobial resistance as well as on molecular relationship of positive and negative isolates are needed to better known the nature of *Campylobacter* pathogenesis. We have made a preliminary study concerning genetic similarity of antimicrobial sensitive and resistant *C. jejuni* using the multilocus sequence typing method (MLST) as described previously (Wieczorek et al., 2017). The results, based on sequence types (STs), demonstrated a high diversity of the isolates in both groups. Strains resistant to ciprofloxacin (*n* = 570) were mainly associated with ST464 (58; 10.2% isolates), ST257 (53; 9.3%), and ST6461 (37; 6.5%) whereas *C. jejuni* sensitive to this antimicrobial (*n* = 47) were classified to other genotypes, e.g., ST583 (6; 12.8% strains), ST122 (4; 8.5%) and ST51 (4; 8.5%). Different STs were also found among tetracycline resistant (ST464; ST257; ST6461) and sensitive isolates (ST50; ST137; ST2036) as well as streptomycin resistant (ST6411; ST6461; ST5397) and sensitive (ST464; ST257; ST50) bacteria tested. A correlation between resistance to tetracycline and quinolones and MLST sequence type 464 among *C. jejuni* isolated from poultry meat was also identified in France (Guyard-Nicodème et al., 2015). Furthermore, it was previously shown that ST464 is more generally associated with quinolone resistance (Wirz et al., 2010). Further broader analyzes are needed for molecular comparison and assessment of association between sequence types, antimicrobial resistance and presence of virulence marker genes among *C. jejuni* isolated from poultry food chain and humans with diarrhea.

## Conclusions

An important step in the prevention and control of campylobacteriosis in humans is identification and characterization of *C. jejuni* that pose the greatest risk to human health, i.e., the isolates which have virulence traits and are resistant to antimicrobials used in treatment of the infection. The goal of this study was to assess the prevalence of markers in *C. jejuni* associated with pathogenesis of the disease and to identify such virulence genes among isolates recovered along poultry food chain. It was shown that strains with crucial pathogenic factors responsible for *C. jejuni* motility (*flaA, flhA*), adherence and colonization (*docA, racR*), toxin production (*cdt*), invasiveness (*ciaB*), and stress response (*sodB*) were highly conserved among isolates of different origin. In contrast, the *virB11, wlaN*, and *iam* were relatively rare and therefore, their role in the pathogenesis of the disease should be further evaluated. It was also found that the majority of *C. jejuni* tested was resistant to ciprofloxacin, nalidixic acid, and tetracycline but they were mostly sensitive to erythromycin and streptomycin. Isolates resistant to quinolones were mostly classified to ST464 subtype as tested by MLST. We have also provided a broad data on the correlation between the presence of key virulence factors and identified interactions between these genes and antimicrobial resistance, especially to macrolides and quinolones. The results of this study show a high prevalence of several pathogenic markers, but it is difficult to predict how virulent or less virulent a particular *C. jejuni* isolate may be *in vivo* during human infection. Therefore, further studies must be performed on the presence or absence of putative pathogenic factors, antimicrobial resistance and molecular relationship among *C. jejuni* food and clinical isolates to provide more information on the pathogenesis of *Campylobacter* infection. Although the exact nature and effects of these two markers for pathogenicity of *C. jejuni* are not yet clear, the results of the present investigations provide a basis for future research important for a public health risk.

## Author contributions

KW and JO conceived the study and contributed material from the poultry chain; TW provided the human *C. jejuni* isolates; KW and JO planned the study; KW and TW performed the experiments; KW and JO analyzed the data and drafted the paper; all authors critically read and approved the final version.

### Conflict of interest statement

The authors declare that the research was conducted in the absence of any commercial or financial relationships that could be construed as a potential conflict of interest.
