# Supplementary material for: Antimicrobial Resistance and Virulence-Associated Traits of Campylobacter jejuni Isolated From Poultry Food Chain and Humans With Diarrhea
Source: Front Microbiol. 2018 Jul 4;9:1508. doi: 10.3389/fmicb.2018.01508 (PMC6039573; doi:10.3389/fmicb.2018.01508)
Supplement: Supplementary file 1 [file Table_1.DOCX]

**Antimicrobial resistance and virulence-associated traits of *Campylobacter jejuni* isolated from poultry food chain and humans with diarrhea**

Kinga Wieczorek, Tomasz Wołkowicz and Jacek Osek

**Supplementary Material**

**TABLE S1. PCR primers and annealing temperatures used for detection of *C. jejuni* virulence genes**

| Virulence trait | Target gene | Sequence (5' - 3') | Annealing temperature | Product size (bp) | Reference |
| --- | --- | --- | --- | --- | --- |
| Motility | *flaA* | F: GGATTTCGTATTAACACAAATGGTGC  R: CTGTAGTAATCTTAAACATTTTG | 48°C | 1,700 | Campynet |
|  | *flhA* | F: GGAAGCGGCACTTGGTTTGC  R: GCTGTGAGTGAGATTATAGCAGC | 55°C | 735 | Müller et al., 2006 |
| Adhesion and colonization | *cadF* | F: TGGAGGGTAATTTAGATATG  R: CTAATACCTAAAGTTGAAAC | 45°C | 400 | Konkel et al., 1997 |
|  | *docA* | F: ATAAGGTGCGGTTTTGGC  R: GTCTTTGCAGTAGATATG | 50°C | 725 | Müller et al., 2006 |
|  | *racR* | F: GATGATCCTGACTTTG  R: TCTCCTATTTTTACCC | 50°C | 584 | Datta et al., 2003 |
|  | *virB11* | F: GAACAGGAAGTGGAAAAACTAGC  R: TTCCGCATTGGGCTATATG | 56°C | 708 | Bacon et al., 2000 |
| Toxin production | *cdtA* | F: CCTTGTGATGCAAGCAATC  R: ACACTCCATTTGCTTTCTG | 55°C | 370 | Hickey et al., 2000 |
|  | *cdtB* | F: CAGAAAGCAAATGGAGTGTT  R: AGCTAAAAGCGGTGGAGTAT | 57°C | 620 | Datta et al., 2003 |
|  | *cdtC* | F: CGATGAGTTAAAACAAAAAGATA  R: TTGGCATTATAGAAAATACAGTT | 55°C | 182 | Datta et al., 2003 |
|  | *wlaN* | F: TGCTGGGTATACAAAGGTTGTG  R: AATTTTGGATATGGGTGGGG | 60°C | 330 | Müller et al., 2006 |
| Invasiveness | *ciaB* | F: TTTCCAAATTTAGATGATGC  R: GTTCTTTAAATTTTTCATAATGC | 50°C | 1,165 | Müller et al., 2006 |
|  | *iam* | F: GCGCAAAATATTATCACCC  R: TTCACGACTACTATGCGG | 56°C | 518 | Carvalho et al., 2001 |
| Stress response | *sodB* | F: ATGATACCAATGCTTTTGGTGATTT  R: TAATACGACTCACTATAGGGCATTTGCATAAAAGCTAACTGATCC | 50°C | 638 | Hanning et al., 2010 |

F, forward; R, reverse

**TABLE S2. Antimicrobials, dilution ranges and cut-off values used in the study.**

| Antimicrobial class | Antimicrobial | Dilution range and cut-off value (mg/L) |
| --- | --- | --- |
| Aminoglycosides | Gentamicin (GEN) | 0.12 – 16; >2 |
|  | Streptomycin (STR) | 1 – 16; >4 |
| Macrolides | Erythromycin (ERY) | 0.5 – 32; >4 |
| Quinolones and fluoroquinolones | Ciprofloxacin (CIP) | 0.06 – 4; >0.5 |
|  | Nalidixic acid (NAL) | 2 – 64; >16 |
| Tetracyclines | Tetracycline | 0.25 – 16; >1 |
